# Development and preliminary clinical validation of A29L mAb-based ELISA and lateral flow immunoassays for monkeypox virus detection

**DOI:** 10.1128/spectrum.00167-26

**Published:** 2026-06-15

**Authors:** Jieguang Liu, Xujuan Hu, Qiong Chen, Xianglian Xiong, Yingjie Zhang, Leyiyi Liu, Si Du, Yuanjun Wu, Dan Fan, Jia Lu, Lianguo Ruan, Fuli Ren

**Affiliations:** 1 Wuhan Jinyintan Hospital, Tongji Medical College of Huazhong University of Science and Technology 66375, Wuhan, Hubei, China; 2 Clinical Biospecimen Resource Center, Wuhan Jinyintan Hospital 194046, Wuhan, Hubei, China; 3 Wuhan Children's Hospital, Tongji Medical College of Huazhong University of Science and Technology 159370, Wuhan, Hubei, China; 4 Wuhan institute of biological products CO., LTD. 155181 , Wuhan, Hubei, China; 5 Hubei Jiangxia Laboratory, Wuhan, Hubei, China; 6 State Key Laboratory for Diagnosis and Treatment of Severe Zoonotic Infectious Diseases, Wuhan, Hubei, China; BBAdvies and Research, Utrecht, the Netherlands; Hubei Jiangxia Laboratory, Wuhan, China; University of Texas Medical Branch at Galveston, Galveston, Texas, USA

**Keywords:** MPXV, A29L protein, monoclonal antibody, ELISA, LFIA

## Abstract

The monkeypox virus (MPXV) poses a significant and ongoing public health threat, highlighting the urgent need for accessible and accurate diagnostic tools. The viral envelope protein A29L is an immunodominant and highly conserved target for immunoassay development. This study aimed to generate a panel of novel monoclonal antibodies (mAbs) against A29L and to develop both a highly sensitive laboratory-based enzyme-linked immunosorbent assay (ELISA) and a rapid lateral flow immunoassay (LFIA) for specific antigen detection. Following immunization and hybridoma screening, six high-affinity mAbs against A29L were selected and fully characterized. Utilizing this antibody panel, a sandwich ELISA was developed, achieving a notably low detection limit of 31.25 pg/mL, with an excellent linear range. In clinical evaluations using samples from suspected MPXV cases (plasma, vesicular fluid, and swabs), the ELISA demonstrated 95% sensitivity and 100% specificity for plasma samples, performing comparably to gold-standard PCR, and outperforming available commercial kits. Furthermore, the developed LFIA demonstrated sensitivities of 58.3% (7/12) in plasma and 40.0% (4/10) in vesicular fluid samples, with perfect detection in high viral load cases, confirming its strong potential for point-of-care deployment. In conclusion, we present a practical, tiered diagnostic strategy that integrates a highly sensitive confirmatory ELISA with a rapid frontline LFIA. This complementary approach effectively bridges the critical gap between centralized confirmatory testing and decentralized field surveillance, significantly enhancing outbreak response capabilities for MPXV.

**IMPORTANCE:**

Monkeypox virus has emerged as a global infectious disease threat, creating an urgent need for accurate and accessible diagnostic tools. Currently, the confirmation of an infection necessitates the execution of sophisticated laboratory tests, which can result in delayed results and impede a swift public health response, particularly in settings characterized by limited resources. This study addresses this critical gap by creating a complete diagnostic toolkit. We developed new, highly specific antibodies against the virus and used them to build two complementary tests: a very sensitive lab-based test for definitive confirmation and a simple, rapid paper-strip test that can be used at the point of care, such as in a clinic or field site. This dual approach provides a practical strategy for improving outbreak control, enabling both accurate laboratory diagnosis and immediate on-site screening to quickly identify infected individuals and help stop the chains of transmission.

## INTRODUCTION

Monkeypox, a disease caused by the monkeypox virus (MPXV), has evolved from a neglected tropical disease into a global public health threat (1). MPXV (genus *Orthopoxvirus*, family *Poxviridae*) was first identified in laboratory monkeys in 1958, with human cases documented since 1970 in Central and West Africa, historically remaining confined to endemic regions (2, 3). The landscape underwent a significant transformation in 2022, when a multinational outbreak prompted the World Health Organization (WHO) to declare a Public Health Emergency of International Concern (PHEIC), highlighting the virus’s ability to facilitate sustained human-to-human transmission and global dissemination (4, 5).

The clinical diagnosis of monkeypox poses a significant challenge due to the non-specificity of its early symptoms, which include fever, lymphadenopathy, and myalgia (6, 7). The rash associated with MPXV can resemble those caused by varicella-zoster virus (VZV) or other poxviruses (8). Although polymerase chain reaction (PCR) detection of viral DNA is the gold standard, its requirement for sophisticated instrumentation, technical expertise, and stable supply chains creates significant bottlenecks in resource-limited settings or during rapid outbreak response, where timely point-of-care testing is critical for containment (9–11).

The lack of rapid tests has spurred interest in developing deployable alternatives such as ELISA and lateral flow immunoassays (LFIA) (12). The successful development of these immunoassays critically depends on the identification of viral antigens that are both highly immunogenic and specific. The MPXV envelope glycoprotein A29L has been identified as a primary candidate for further study. It is a conserved, structurally essential protein involved in host cell attachment and entry, and it elicits a potent antibody response during infection (13–15). While the protein’s homology with other orthopoxvirus proteins necessitates careful epitope selection to ensure MPXV specificity, its consistent expression makes it a reliable diagnostic target (16–19). Monoclonal antibodies (mAbs), with their well-defined specificity and ample production capacity, are the optimal reagents to fully realize the diagnostic potential of A29L (20, 21). Therefore, to bridge this critical diagnostic gap, a panel of novel murine mAbs against A29L was generated, and then these mAbs were leveraged to develop two complementary platforms for robust antigen detection: a sensitive ELISA and a rapid LFIA.

In the present study, we developed and optimized two diagnostic platforms by leveraging high-affinity and specific mAbs as core reagents. The first platform is a highly sensitive sandwich ELISA for laboratory use, and the second is a rapid colloidal gold-based LFIA for point-of-care testing. The analytical performance and clinical utility of both assays were rigorously evaluated using well-characterized specimens. This work provides a comprehensive immunoassay toolkit for MPXV, thereby establishing a foundation for accessible and timely diagnosis in both clinical and public health contexts.

## MATERIALS AND METHODS

### Plasmids, cell line, and animals

The mouse myeloma cell line SP2/0 was obtained from the Wuhan Institute of Biological Products Co., Ltd. The cells were cultivated at 37°C in a 5% CO_2_ atmosphere in RPMI 1640 medium, with the following supplements: 2 mM L-glutamine, 100 U/mL penicillin, 100 μg/mL streptomycin, and 10% heat-inactivated fetal bovine serum (FBS). A recombinant plasmid expressing MPXV A29L as a fusion protein with glutathione S-transferase (GST) was constructed using the pGEX-4T-1 vector. Eight-week-old specific pathogen-free (SPF) female Balb/c mice were supplied by the same institute and housed under standard conditions.

### Virus, antigens, and clinical specimens

The MPXV (WIBP-MPXV-001), which belongs to clade II, was isolated from clinical specimens in a biosafety level 3 laboratory at the Wuhan Institute of Biological Products Co., Ltd. The MPXV was propagated in Vero E6 cells in DMEM, with the addition of 2% newborn bovine serum (NBS) and 50 units/mL penicillin–streptomycin, as previously reported by the authors (22).

The commercial recombinant MPXV A29L proteins from clade II (Cat# 40891-V08E) and clade Ib (Cat# 41041-V08E), the Monkeypox Virus (MPXV) A29L ELISA Kit (Cat# KIT40891), and an anti-MPXV A29L monoclonal antibody (Cat# 40891-MM17) were commercially sourced from Sino Biological Inc. (Beijing, China). The A27L antigen of Vaccinia Virus (VACV, Cat# P100150) was provided by Zoonogen Biological Inc. (Beijing, China). A series of clinical specimens were collected at Wuhan Jinyintan Hospital, with appropriate ethical approval and patient consent. These specimens included human plasma, vesicle fluid, and anal/pharyngeal swabs from suspected MPXV cases and healthy volunteers.

### Recombinant protein production and animal immunization

The pGEX-4T-1-A29L plasmid expressing full-length A29L protein was transformed into *E. coli* TOP10 cells. The induction of protein expression was achieved by employing 0.5 mM isopropyl β-D-1-thiogalactopyranoside (IPTG). The A29L-GST fusion protein was purified using glutathione-Sepharose 4B affinity chromatography (Cat# 17075605, Cytiva, USA) and served as the immunogen.

Five female Balb/c mice were subcutaneously immunized with 50 μg of purified A29L-GST emulsified in Freund’s complete adjuvant (Cat# AR001, Sigma-Aldrich, USA). Subsequent to this, three booster immunizations were administered at 14-day intervals, employing 25 μg of antigen emulsified in Freund’s incomplete adjuvant (Cat# AR002, Sigma-Aldrich, USA). A final intraperitoneal boost of 15 μg of antigen in phosphate-buffered saline (PBS) was administered 3 days prior to cell fusion.

### Generation and characterization of monoclonal antibodies

The generation of hybridomas was achieved through the process of electrofusion, whereby SP2/0 cells and splenocytes from immunized mice were fused at a 1:10 ratio, in accordance with a well-established protocol (23). The screening of the supernates was conducted through the utilization of an indirect ELISA, with purified A29L serving as the coating antigen. Positive clones were subcloned in triplicate by means of limiting dilution to ensure a single monoclonal population.

The isotype of each mAb was determined using a commercial Mouse Monoclonal Antibody Isotyping Kit (Cat# PK20002, Proteintech, USA). Antibody sequencing services were provided by AtaGenix Biotechnology Co., Ltd. (Wuhan, China).

### SDS-PAGE and Western blot analysis

Protein samples were denatured in 5× loading buffer, separated on 10% SDS-PAGE gels, and visualized by Coomassie Brilliant Blue R-250 staining. For immunoblotting, proteins were transferred onto PVDF membranes (Cat# 29301-808, Pall Corporation, USA). Membranes were blocked with 5% skim milk in PBST (PBS with 0.05% Tween-20), incubated with either the commercial anti-A29L antibody or the prepared mAbs (1:1,000 dilution), followed by incubation with an HRP-conjugated goat anti-mouse IgG (H + L) secondary antibody (Cat# SA00001-1, Proteintech, USA). Signals were developed using a DAB substrate kit (Cat# 34002, Thermo Fisher Scientific, USA). Lysate from empty vector-transformed *E.coli* served as the negative control.

### Virus neutralization test (VNT)

Neutralization assays were performed in a biosafety level 3 (BSL-3) laboratory. For the plaque reduction neutralization test (PRNT), serial two-fold dilutions of serum or purified mAbs were mixed with an equal volume of MPXV, titrated to approximately 150 plaque-forming units (PFU) per well, and incubated at 37 °C for 1 h. The mixtures were added to confluent (>90%) monolayers of MDBK cells in 24-well plates. After a 1-hour adsorption period, the inoculum was replaced with an overlay medium (EMEM containing 2% FBS and 0.9% methylcellulose). Plates were incubated for 48–72 h, fixed with 4% paraformaldehyde, and stained with 0.05% crystal violet. Each assay included virus-only (no antibody) controls and cell-only controls. The neutralizing antibody titer (PRNT₅₀) was defined as the highest antibody dilution that reduced the number of plaques by ≥50% compared to the virus-only control wells. Samples exhibiting plaque reduction levels below 50% at the initial dilution (1:4) were designated as negative.

The half-maximal inhibitory concentration (IC₅₀) was calculated by fitting the dose-response curve of plaque reduction data using a four-parameter logistic model in GraphPad Prism (version 8.0).

### Preparation, biotinylation, and pairwise screening of mAbs

Prior to the assembly of the assay, the six candidate monoclonal antibodies were produced in bulk via hybridoma culture and purified using Protein G affinity chromatography. The antigen-binding titers of all purified mAbs, as assessed by indirect ELISA with immobilized A29L antigen (2.0 µg/mL), were confirmed to be sufficiently high for the subsequent stages of development. In order to ensure compatibility with a streptavidin-based detection system, each purified mAb was conjugated with biotin via amine coupling, thereby generating the derivatives Bio-5, Bio-25, Bio-28, Bio-87, Bio-94, and Bio-111. Indirect ELISA was employed to confirm that biotinylation did not compromise antigen-binding activity, as all conjugated antibodies retained high reactivity.

A systematic, pair-wise screening was performed with the objective of identifying the optimal antibody pair for a sandwich ELISA. Checkerboard assays were employed, whereby various capture antibodies (1.0–5.0 μg/mL coating concentration) were paired with biotinylated detection antibodies (0.1–1.0 μg/mL) across a range of recombinant A29L antigen concentrations (1.56–100 ng/mL). The combination yielding the highest signal-to-noise ratio was selected for subsequent assay development and optimization. The optimal working concentrations for the selected antibody pair were determined using a refined checkerboard titration.

### Development of the sandwich ELISA

An enzyme-linked immunosorbent assay (ELISA) has been developed for the specific detection of the MPXV A29L antigen. High-binding 96-well microplates were coated overnight at 4°C with 100 µL per well of the selected capture antibody (mAb#94), which had been diluted in 0.05 M carbonate-bicarbonate buffer (pH 9.6). The optimal coating concentration (ranging 1.0–5.0 µg/mL) was determined by checkerboard titration.

Following the coating step, the plates were washed thrice with PBST (PBS containing 0.05% Tween-20). Thereafter, they were blocked with 250 µL per well of a blocking buffer (3% [wt/vol] bovine serum albumin [BSA] in PBS) for 1.5 h at 37°C to prevent non-specific binding. Following a subsequent washing step, 100 µL of either serial dilutions of recombinant A29L antigen or processed clinical samples (diluted in sample dilution buffer: 1% [wt/vol] BSA in PBS) were added to the wells and incubated for 1.0 h at 37°C. The plates were washed again, and 100 µL of a different, biotinylated anti-A29L monoclonal antibody (another selected mAb from the panel, biotinylated using the EZ-Link Sulfo-NHS-LC-Biotinylation Kit [Cat# 21435, Thermo Scientific, USA] according to the manufacturer’s instructions) was added at its predetermined optimal concentration and incubated for 1.0 h at 37°C. Subsequent to a wash step, 100 µL of streptavidin-horseradish peroxidase (HRP) conjugate (Cat# SA10001, Thermo Scientific, USA), diluted in accordance with the manufacturer’s instructions, was added and incubated for 30 min at 37°C.

Following a final wash, the enzymatic reaction was initiated by the addition of 100 µL of Invitrogen TMB Chromogen Solution (Cat# 00-202-3, Invitrogen, USA) and incubation for 10 min in the dark at room temperature. The reaction was then halted by the addition of 50 µL of 2.0 M H₂SO₄, and the resulting change in optical density was immediately measured at a wavelength of 450 nm (reference wavelength: 620 nm) using a microplate reader (BioTek Instruments, Agilent, Model: Synergy H1). A standard curve was plotted from the recombinant antigen dilutions. Based on this standard curve, the assay demonstrated a linear quantitative range from 31.25 to 2,000 pg/mL. The limit of detection (LOD) and limit of quantification (LOQ) were determined to be 31.25 pg/mL and 62.50 pg/mL, respectively. The LOD was defined as the lowest antigen concentration yielding a signal significantly above the blank (mean + 3 standard deviations), and the LOQ was defined as the lowest concentration measurable with acceptable precision (coefficient of variation < 20%) and accuracy (80–120% recovery). The concentration of A29L in unknown samples was interpolated from this curve. Each assay incorporated blank wells (no antigen), negative control wells (coating antibody + sample dilution buffer), and positive control wells (coating antibody + antigen) for quality assurance.

### Development of the LFIA

The colloidal gold nanoparticles under scrutiny were prepared and characterized by Zoonbio Biotechnology Co., Ltd. (Nanjing, China). In summary, 1.0 mL of colloidal gold solution was subjected to pH adjustment with 0.2 M K_2_CO_3_. Subsequently, 10 µg of purified anti-A29L mAb was added and allowed to conjugate for 30 min at room temperature. The conjugation process was stabilized through the use of a blocking agent, 50 μL of 10% BSA, which was applied for 30 min. The conjugate was pelleted by means of centrifugation at 11,000 × *g* for 20 min, following which it was resuspended in 0.5 mL of preservation buffer (comprising 1% BSA, 5% sucrose, and 0.1% sodium azide in 10 mM PBS, pH 7.4). Thereafter, the conjugate was sprayed onto a glass fiber pad to prepare the conjugate pad.

A second anti-A29L mAb (1.0 mg/mL in phosphate buffer) and goat anti-mouse IgG (1.0 mg/mL) were dispensed onto a nitrocellulose membrane (Cat# CN140, Sartorius, Germany) to form the test and control lines, respectively. The membrane, conjugate pad, sample pad, and absorbent pad were then assembled and laminated onto a backing card. The card was subjected to drying conditions at a temperature of 37°C for a duration of 12 h, subsequently followed by division of the card into strips measuring 3 mm in width using a precision cutting apparatus. The strips were stored desiccated at ambient temperature until use.

## RESULTS

### Recombinant antigen production, immunization, and hybridoma generation

**Fig 1:**
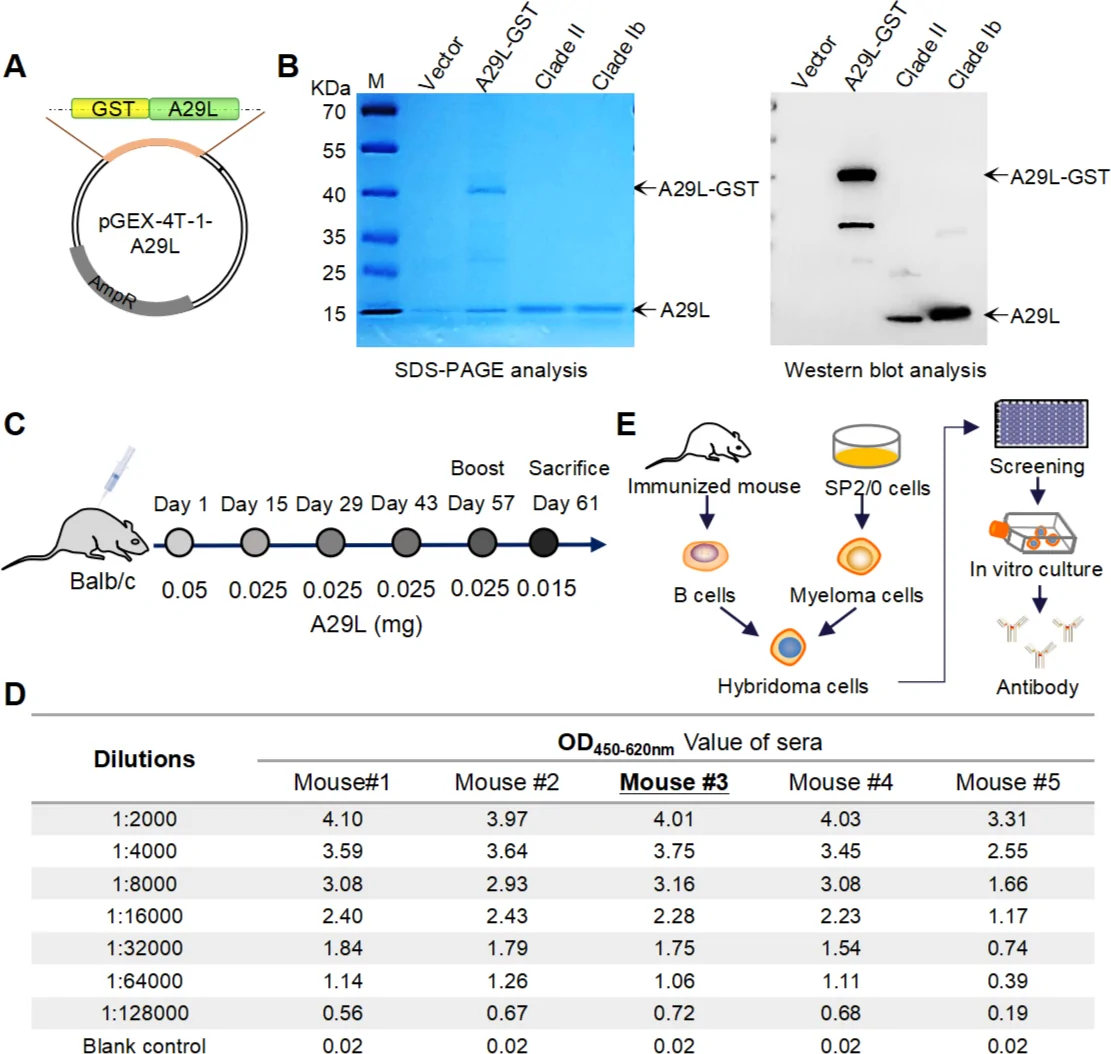
Generation and analysis of hybridoma cells for the production of monoclonal antibody specific to the MPXV A29L antigen. (**A**) Construction strategy of plasmid for expressing A29L-GST fusion protein. (**B**) SDS-PAGE (left) and Western blot (right) analysis of MPXV A29L-GST fusion protein with A29L antigen of clade II and clade Ib as controls. (**C**) Animal vaccination strategy and schedule. (**D**) Titer estimation of antibodies to MPXV in the sera from immunized animals by indirect ELISA. The underline and bold indicate that the selected mouse was used for the next hybridoma cell fusion assay. (**E**) Schematic of the hybridoma cell fusion process and monoclonal antibody production.

To generate a high-quality immunogen and reference standard, the full-length MPXV A29L gene was synthesized and cloned into the pGEX-4T-1 expression vector (Fig. 1A). The resulting construct was transformed into *E. coli* for recombinant protein production. Following IPTG induction, a soluble A29L-GST fusion protein of approximately 40 kDa was successfully expressed and purified via glutathione affinity chromatography. The SDS-PAGE analysis confirmed the high purity of the preparation, as evidenced by the presence of a single predominant band at the expected molecular weight. The A29L-GST fusion protein band was observed in the purified product but was absent in the control lysates from empty vector-transformed bacteria (Fig. 1B, left panel). The identity and immunoreactivity of the purified protein were further verified by Western blot using a commercial anti-A29L antibody, which recognized a single specific band corresponding to the A29L-GST fusion protein (Fig. 1B, right panel).

This purified A29L-GST protein was subsequently used to immunize BALB/c mice according to the regimen detailed in Fig. 1C. The immunization strategy comprised a primary subcutaneous injection with antigen emulsified in Freund’s complete adjuvant, followed by multiple booster injections of antigen in incomplete Freund’s adjuvant. Sera collected from immunized mice were evaluated by indirect ELISA, which demonstrated a significant and robust antibody response in all five mice, with high endpoint titers against the A29L antigen (Fig. 1D). The robust serum antibody response confirmed the immunogenicity of the recombinant antigen and supported the progression to hybridoma generation.

Splenocytes from the immunized mouse showing the highest antibody titer were fused with SP2/0 myeloma cells (Fig. 1E). Subsequent HAT selection was applied to eliminate unfused myeloma cells and permit selective growth of hybridomas.

### Selection and biochemical characterization of anti-A29L mAbs

**Fig 2:**
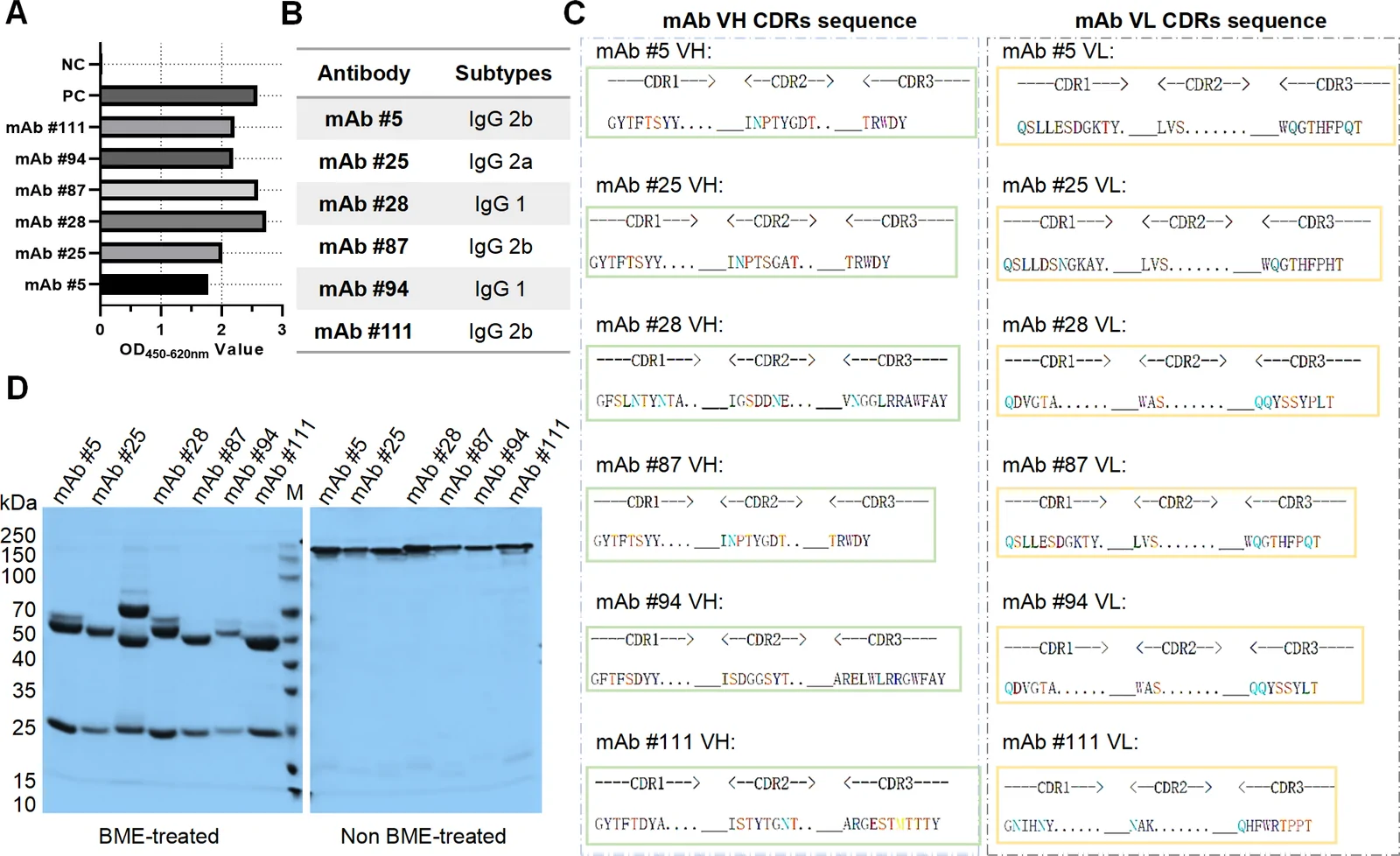
Validation and analysis of monoclonal antibodies specific to MPXV A29L antigen. (**A**) Titer analysis of the supernatant of six subclonal hybridoma cells cultured *in vitro* which secreted antibodies to the MPXV A29L antigen. (**B**) Isotype identification of six monoclonal antibodies. (**C**) Heavy-chain (VH) and light-chain (VL) CDRs sequence analysis of six monoclonal antibodies including mAb#5, #25, #28, #94, and #111. (**D**) BME-treated and non-BME-treated SDS-PAGE analysis of six monoclonal antibodies.

Hybridoma supernatants from over 200 primary clones were screened by indirect ELISA using purified A29L as the coating antigen. Six hybridoma cell lines—designated MPXV-mAb#5, #25, #28, #87, #94, and #111—stably secreting antibodies with high reactivity to A29L were selected for further expansion and subcloning by limiting dilution (Fig. 2A).

The immunoglobulin isotypes of these monoclonal antibodies were determined. The panel comprised two IgG1/κ, one IgG2a/κ, and three IgG2b/κ antibodies (Fig. 2B). To gain deeper insight into the molecular characteristics of these clones, the variable regions of the heavy and light chains (VH and VL) from five of the mAbs were sequenced. Analysis of the complementarity-determining regions (CDRs) revealed unique amino acid sequences for each clone, providing molecular confirmation of their distinct clonal origins and specific binding to the A29L protein (Fig. 2C).

The purified monoclonal antibodies were analyzed by SDS-PAGE under reducing and non-reducing conditions. Under non-reducing conditions, all five antibodies exhibited a molecular mass of approximately 150 kDa, consistent with intact immunoglobulin molecules. Under reducing conditions, they dissociated into heavy chains (~50 kDa) and light chains (~25 kDa). These results confirm the proper assembly and disulfide bonding of the antibodies (Fig. 2D).

### Neutralization activity of Anti-A29L mAbs

To evaluate the virus-neutralizing potential of the six purified monoclonal antibodies (mAb#5, #25, #28, #87, #94, #111), a PRNT was conducted under biosafety level 3 (BSL-3) conditions. The results demonstrated that mAb#5, #28, #87, #94, and #111 exhibited no detectable neutralization at the concentrations tested (IC₅₀ > 0.10 mg/mL). In contrast, mAb#25 showed moderate neutralizing activity, with an IC₅₀ of 0.025 mg/mL (173 nM). This neutralizing potency, while moderate compared to some highly potent therapeutic antibodies against other viruses, suggests that mAb#25 may recognize a functionally important epitope on A29L involved in viral entry.

Although the neutralizing activity of mAb#25 warrants further investigation for potential therapeutic applications, the primary objective of this study was to exploit the high affinity and specificity common to all generated mAbs for the development of diagnostic reagents.

### Analytical performance of the optimized sandwich ELISA

The analytical performance of the optimized sandwich ELISA was rigorously characterized. All six mAbs were produced, purified, biotinylated, and confirmed to retain high antigen-binding activity. Following initial screening of antibody pairs (Tables S1 and S3), the combination mAb#94/Bio-5 was identified as the optimal pairing, demonstrating the highest analytical sensitivity (Table 1). The optimal working concentrations for the coating and detection antibodies were subsequently determined (Table S4). Using a serial dilution of recombinant A29L, the assay demonstrated a linear quantitative range from 31.25 to 2000 pg/mL (Table 2). The LOD, defined as the antigen concentration yielding a signal significantly above the cutoff (*P*/*N* ≥ 2), was 31.25 pg/mL, and the limit of quantification (LOQ) was established at 62.50 pg/mL (*P*/*N* ≥ 5). The assay exhibited excellent reproducibility, with intra- and inter-assay coefficients of variation below 8% and 12%, respectively.

**TABLE 1:** Analysis of monoclonal antibody pairing^*a*^

MPXV A29LDilution (ng/ml)	mAb #28	mAb #28	mAb #94
	Bio-5	Bio-94	Bio-5
100	4.30	4.59	4.80
50^*b*^	4.55	4.72	4.68
25	4.46	4.49	4.41
12.5	4.74	3.21	**4.85**
6.25	4.59	1.60	4.67
3.125	3.06	0.75	3.73
1.5625	1.79	0.36	2.52
0	0.13	0.14	0.16

^*a*^ The underline and bold indicate that the coating antibody and enzyme-labeled antibody corresponding to this value are selected as the best paired antibodies. The optimized working concentrations were 2.0 μg/mL for the coating antibody and 0.2 μg/mL for the enzyme-labeled antibody.

^*b*^ Grey shading is used solely for visual distinction and layout purposes.

**TABLE 2:** Analytical sensitivity of the optimized ELISA^*a*^

MPXV A29L dilution (pg/mL)	mAb#94 (2 μg/mL)
	Bio-5 (0.1 μg/mL)
2000	2.54
1000	1.91
500	1.12
250^*b*^	0.54
125	0.35
62.5	0.23
31.25	0.14
0	0.05

^*a*^ The optimized working concentrations were 2.0 μg/mL for the coating antibody and 0.2 μg/mL for the enzyme-labeled antibody.

^*b*^ Grey shading is used solely for visual distinction and layout purposes.

**Fig 3:**
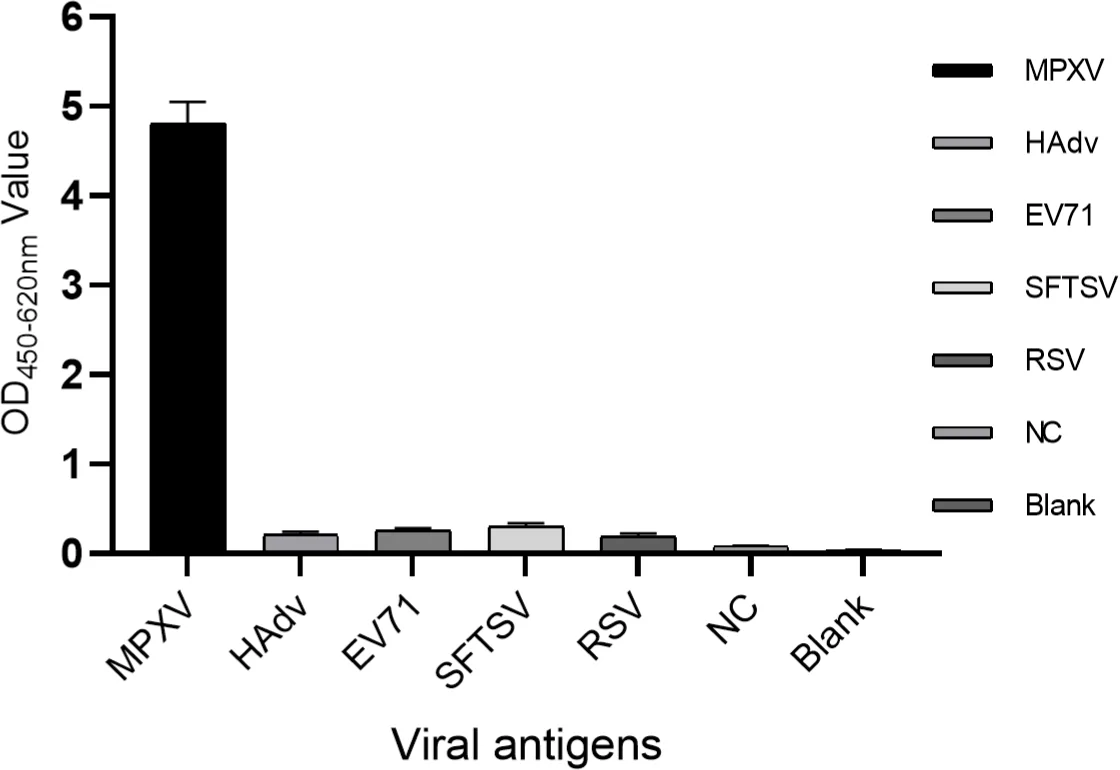
Specificity analysis of the optimized sandwich ELISA. The assay’s cross-reactivity was evaluated against a panel of heterologous viral antigens, including vaccinia virus (VACV), varicella-zoster virus (VZV), herpes simplex virus (HSV), human adenovirus (HAdV), enterovirus 71 (EV71), severe fever with thrombocytopenia syndrome virus (SFTSV), and respiratory syncytial virus (RSV). The optical density (OD_450–620nm_) signal generated by the MPXV A29L antigen (positive control) and virus dilution solution (NC, negative control) are shown for comparisons. Data are presented as mean ± SD from triplicate measurements.

Specificity was also evaluated against a panel of unrelated viral antigens, including vaccinia virus (VACV), varicella-zoster virus (VZV), herpes simplex virus (HSV), human adenovirus (HAdV), enterovirus 71 (EV71), severe fever with thrombocytopenia syndrome virus (SFTSV), and respiratory syncytial virus (RSV). The mAb#94/Bio-5-based ELISA detected only MPXV A29L with high signal, showing no appreciable cross-reactivity with any of the heterologous antigens, thereby confirming its high specificity (Fig. 3).

### Comparison and selection of LFIA configurations

In order to translate this high-performance antibody pair into a format suitable for point-of-care testing, a colloidal gold-based LFIA was developed. Two distinct antibody configurations were evaluated for the LFIA: (a) MPXV-mAb#5 on the test line with MPXV-mAb#94 conjugated to gold nanoparticles and (b) MPXV-mAb#94 on the test line with MPXV-mAb#5 as the conjugate. In comparison with configuration a, configuration b (capture: mAb#94, detector: mAb#5-gold) exhibited superior analytical sensitivity.

**Fig 4:**
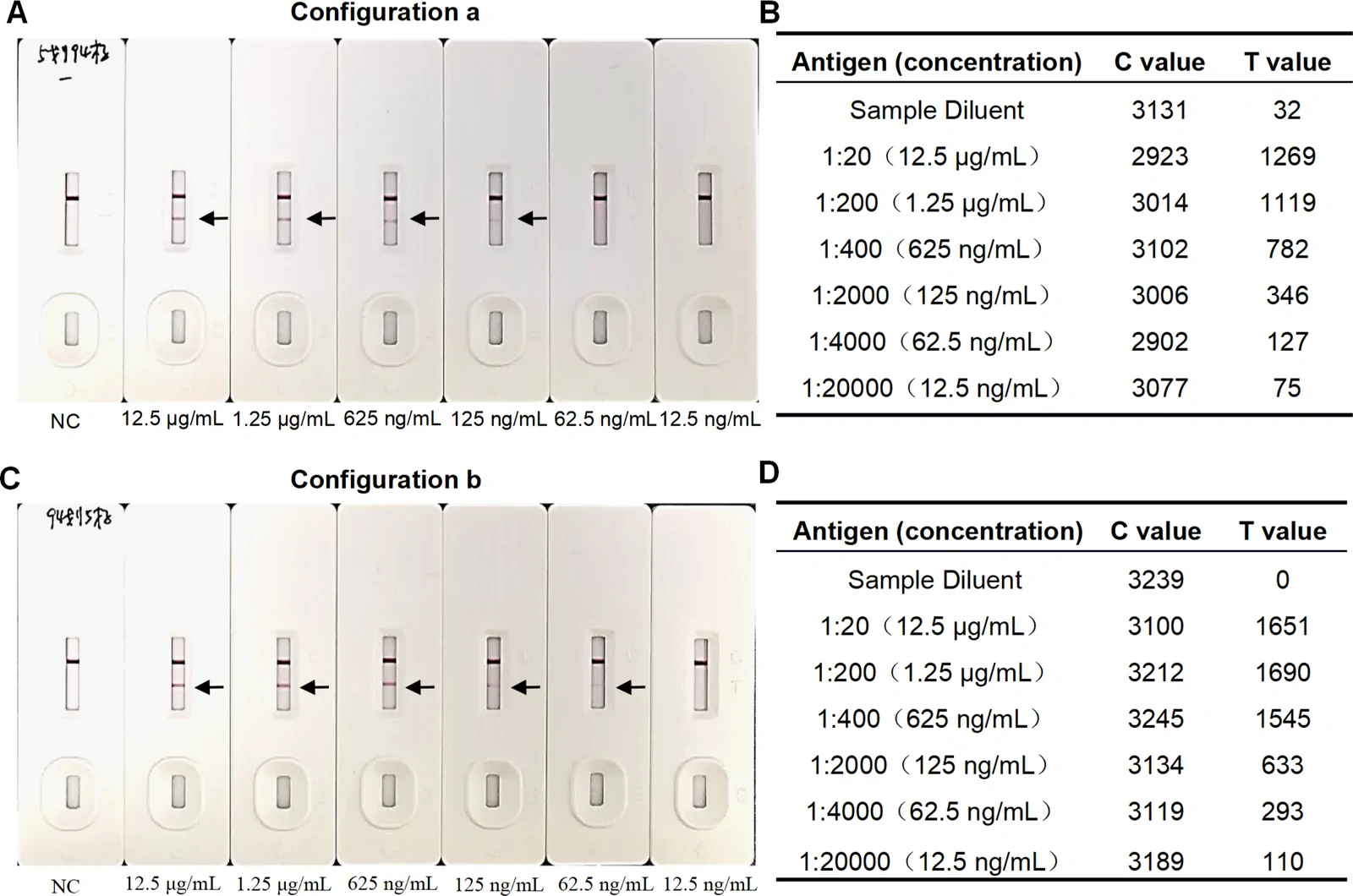
Analytical sensitivity evaluation of two LFIA configurations. Analysis of configuration a, which used mAb#5 as the capture antibody on the test line and mAb#94 conjugated to colloidal gold nanoparticles as the detector. (**A**) Representative strips showing test (T) and control (**C**) line signals in response to a serial dilution series of recombinant A29L antigen (starting concentration: 250 μg/mL). The T-line signals are indicated by black arrows. (**B**) Quantitative analysis of the C- and T-line intensities from the strips in panel **A**. (**C and D**) Analysis of configuration b, which used MPXV-mAb#94 as the capture antibody on the test line and MPXV-mAb#5 conjugated to colloidal gold as the detector. (**C**) Representative strips and (**D**) their corresponding quantitative line intensities for the same antigen dilution series. Configuration b demonstrated superior analytical sensitivity, with a visible T-line at a higher dilution than configuration a.

Configuration b produced a visible test line at an antigen concentration of 62.5 ng/mL and a faint band as low as 12.5 ng/mL (Fig. 4C and D). In contrast, configuration a required a higher antigen concentration (125 ng/mL) to generate a clear visible signal (Fig. 4A and B). Quantitative analysis using a fluorescent immunochromatographic reader (BFIC-Q1, Big Fish, China) corroborated these visual observations (Fig. 4B and D). Consequently, configuration b was selected for further clinical evaluation.

### Clinical evaluation of the ELISA and LFIA

The clinical performance of the two complementary platforms—the laboratory-based ELISA and the point-of-care LFIA—was assessed using well-characterized specimens, with qPCR as the reference method. The sandwich ELISA was tested on matched patient samples of plasma, vesicular fluid, and pharyngeal swabs. Using qPCR as the reference standard, the ELISA demonstrated sensitivities of 100% (10/10) in plasma, 40% (4/10) in vesicular fluid, and 0% (0/10) in pharyngeal swabs (Table 3), markedly outperforming a commercial kit (≤10% detection across all sample types; Table S5).

**TABLE 3:** Clinical evaluation of the ELISA using patient samples^*a*^

Sample	OD450-640nm value		
	Plasma	Pharyngeal swab	Acne liquid
Sample#1	**1.76**	0.04	0.06
Sample#2	**3.02**	0.04	0.02
Sample#3	**2.08**	0.1	0.09
Sample#4	**1.3**	0.08	**0.16**
Sample#5	**0.98**	0.11	0.04
Sample#6	**1.07**	0.09	**0.28**
Sample#7	**1.7**	0.02	**0.14**
Sample#8	**1.51**	0.11	**1.44**
Sample#9	**3.6**	0.01	0.07
Sample#10	**0.61**	0.09	0.01
Positive control	**2.16**	**1.84**	**1.69**
Negative control	0.04	0.07	0.06

^*a*^ Samples were considered positive when indicated by underlined and bolded values. Recombinant A29L antigen was used as the positive control. Negative controls consisted of samples from healthy donors for plasma and pharyngeal swabs and PBS for vesicular fluid. *P*/*N* value ≥ 2.1 was defined as the threshold for positivity.

**Fig 5:**
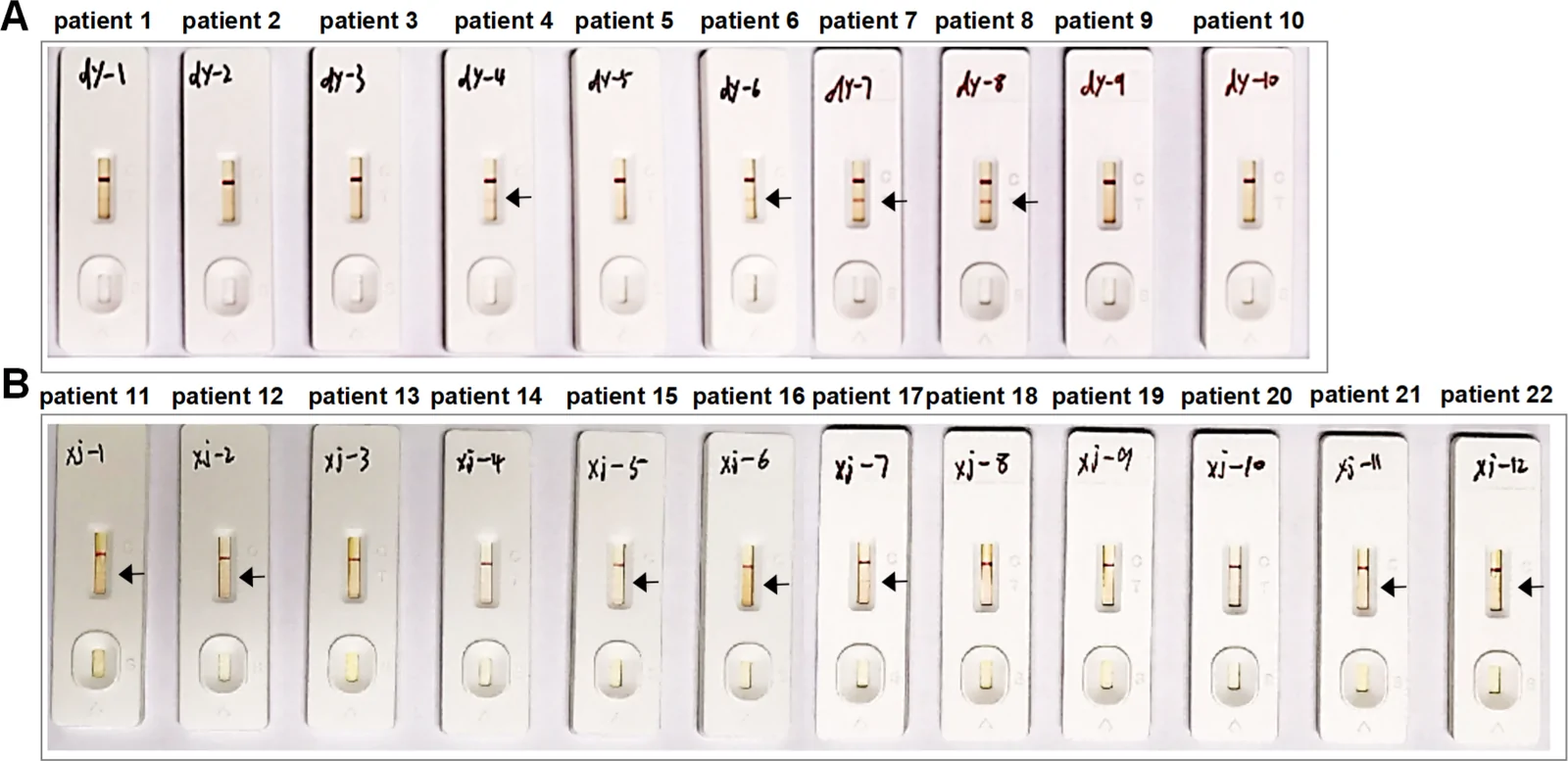
Clinical evaluation of the LFIA using patient samples (**A**) Representative test strips showing results obtained with vesicular fluid samples from clinically confirmed MPXV patients (*n* = 10) using configuration b (capture antibody: MPXV-mAb#5; detector: mAb#94-gold conjugate). The test (T) lines are indicated with black arrows (*n* = 10). The T-line signals are indicated by black arrows. (**B**) Representative test strips of plasma samples from confirmed MPXV patients (*n* = 12) tested under the same configuration. Black arrows indicate visible T-lines.

The LFIA was evaluated using 22 clinical samples (12 plasma, 10 vesicular fluid). The LFIA detected MPXV antigen in 7/12 plasma and 4/10 vesicular fluid samples (Fig. 5B and C, showing representative test strips for plasma and vesicular fluid, respectively). Notably, in the subset of samples with high viral loads (qPCR Ct < 30), the LFIA demonstrated a sensitivity of 100%. The corresponding qPCR Ct values for all LFIA-tested samples are detailed in Table S6. The performance data from both assays validate a tiered diagnostic strategy: the ELISA serves as a highly sensitive confirmatory tool in clinical laboratories, while the LFIA provides a rapid, equipment-free screening option particularly effective in identifying individuals with high viral loads, who are most contagious.

## DISCUSSION

The global spread of MPXV has exposed a critical diagnostic gap, highlighting an urgent need for accurate tests deployable outside central laboratories (3, 24, 25). In addressing the need for field-deployable diagnostics, we adopted a systematic approach commencing with the generation of novel mAbs directed against the immunodominant A29L protein and culminating in the development of two complementary diagnostic platforms.

The generation of six high-affinity mAbs against A29L has been demonstrated to be a success, thus emphasizing the strong immunogenicity of this viral protein, which is consistent with the critical role of A29L in viral entry and pathogenesis. Epitope binning analysis via competitive ELISA revealed that the six mAbs could be categorized into distinct groups, confirming the presence of multiple non-overlapping antigenic sites on A29L (26, 27). The identification of non-overlapping epitopes was pivotal, as it enabled the selection of the mAb#94/Bio-5 pair, which has the capacity to bind to distinct epitopes in a simultaneous manner. Pairing antibodies that bind to distinct, non-overlapping epitopes minimizes steric hindrance, maximizing antigen capture and detection efficiency—a fundamental reason behind the exceptional sensitivity (LOD: 31.25 pg/mL) of our sandwich ELISA, which surpasses that of previously reported immunoassays for MPXV (28). The negligible cross-reactivity observed reinforces the high specificity of our antibodies for MPXV A29L, a crucial attribute for avoiding false positives in clinical settings where other poxviruses or unrelated febrile illnesses may be present.

In addition to the laboratory-based ELISA, we were instrumental in translating these reagents into a format that was immediately applicable to public health: the LFIA. The development of a rapid test is not merely a technical exercise but a strategic response to the realities of outbreak containment (29). While PCR remains the irreplaceable gold standard for its sensitivity, its reliance on sophisticated infrastructure and time-intensive protocols creates a diagnostic vacuum during the critical early phases of an outbreak (30). The LFIA test, which exhibits strong concordance with PCR in high viral load samples and requires minimal training to operate, is designed to fill this gap. The LFIA is a potent instrument for the expeditious screening, triage, and field-based surveillance of patients, thereby enabling immediate isolation decisions and contact tracing in settings where resources are limited. We propose that this rapid screening capability could significantly curtail transmission chains.

The LFIA, while exhibiting a higher analytical detection limit than the ELISA, fulfills a distinct and critical public health role. Its performance profile aligns with the “detect-to-protect” paradigm in outbreak settings. The high specificity of the test minimizes the occurrence of false alarms, and its capacity to accurately identify individuals with high viral loads (Ct < 30)—who are likely to be the most infectious—enables expeditious triage, isolation, and contact tracing at the point of need. This can definitively disrupt transmission chains in community or resource-constrained contexts, thereby addressing the diagnostic gap that exists prior to the availability of PCR results. Consequently, our LFIA can be seamlessly integrated into a tiered testing algorithm, serving as a rapid rule-in test at points of entry, in community clinics, or during outbreak investigations to immediately isolate likely infectious cases, while samples are forwarded for confirmatory PCR testing. This dual-platform approach, comprising an ultrasensitive ELISA for confirmatory testing and a rapid LFIA for frontline screening, offers a holistic and tiered diagnostic strategy.

A critical discussion point is the performance of our assays against commercially available kits. The sensitivity and specificity of the in-house ELISA exceeded those of the commercially available Monkeypox Virus (MPXV) A29 ELISA Kit (Cat# KIT40891, SinoBiological) in parallel testing. This superior performance underscores the value of *de novo* development of tailored, high-affinity antibody pairs, which can yield more robust antigen detection systems than relying on generic commercial reagents. This highlights the significance of fundamental reagent development as the foundation for superior diagnostic products, thereby ensuring independence from commercially available antibodies that may be suboptimal.

The impact of this study is constrained by the preliminary nature of the clinical evaluation. The performance of the ELISA and LFIA was assessed using a limited, single-center cohort from Hubei Province, China (*n* = 22 for LFIA; all clade IIb). Therefore, future studies with larger, geographically diverse samples, including other MPXV clades, are warranted to confirm broad applicability. Additionally, the long-term stability of the LFIA strips under field conditions warrants further investigation. Finally, ongoing surveillance of A29L sequence evolution is essential—as mutations in the targeted epitopes could potentially affect assay efficacy over time. However, our sequence conservation analysis confirmed a high degree of amino acid identity (>99%) among the major MPXV clades (I, IIa, and IIb). This strong conservation supports the potential broad applicability of the developed antibodies across different viral lineages and suggests a relatively low risk of near-term diagnostic escape. To ultimately translate these assays into widespread clinical use, future multicenter studies with larger sample sizes are required to validate their utility across diverse populations and MPXV clades.

In conclusion, we have delivered not merely two diagnostic assays, but a validated workflow. This workflow encompasses target characterization, high-quality mAb generation, and the development of both laboratory and point-of-care detection platforms. This integrated approach provides a robust blueprint for rapidly creating effective diagnostic solutions against emerging pathogens, thereby enhancing our preparedness for future public health threats.

## Supplementary Material

Reviewer comments

## Data Availability

The datasets generated and analyzed during this study are not publicly available due to patient privacy concerns but are available from the corresponding author (F.L. Ren) upon reasonable request and subject to a data use agreement.

## SUPPLEMENTAL MATERIAL

Supplemental tables spectrum.00167-26-s0001.docx Tables S1 to S6.

OPEN PEER REVIEW reviewer-comments.pdf An accounting of the reviewer comments and feedback.

ASM does not own the copyrights to Supplemental Material that may be linked to, or accessed through, an article. The authors have granted ASM a non-exclusive, world-wide license to publish the Supplemental Material files. Please contact the corresponding author directly for reuse.
