# Supplementary material for: Development and preliminary clinical validation of A29L mAb-based ELISA and lateral flow immunoassays for monkeypox virus detection
Source: Microbiol Spectr. 2026 Jun 15;14(7):e00167-26. doi: 10.1128/spectrum.00167-26 (PMC13340043; doi:10.1128/spectrum.00167-26)
Supplement: Supplemental tables — Tables S1 to S6. [file spectrum.00167-26-s0001.docx]

**Supplementary TABLES and FIGURES:**

**TABLE S1**.**Titers analysis of anti-A29L monoclonal antibodies**.

| **Dilution** | **OD_450-620nm_ Value** | | | | | |
| --- | --- | --- | --- | --- | --- | --- |
|  | **mAb #5** | **mAb #25** | **mAb #28** | **mAb #87** | **mAb #94** | **mAb #111** |
| 1:2000 | 3.40 | 3.00 | 2.77 | 3.42 | 3.64 | 3.52 |
| 1:4000 | 3.02 | 2.45 | 1.92 | 3.15 | 2.93 | 3.34 |
| 1:8000 | 2.91 | 2.10 | 1.32 | 2.90 | 2.63 | 3.36 |
| 1:16000 | 2.45 | 1.60 | 1.08 | 2.76 | 2.24 | 3.00 |
| 1:32000 | 2.24 | 1.22 | 0.65 | 2.37 | 1.62 | 2.90 |
| 1:64000 | 1.96 | 0.95 | 0.48 | 2.18 | 1.21 | 2.62 |
| 1:128000 | 1.58 | 0.61 | 0.33 | 1.90 | 0.82 | 2.17 |
| Blank control | 0.04 | 0.04 | 0.04 | 0.04 | 0.04 | 0.04 |

Note: Antibodies were produced on a large scale from six hybridoma clones (#5, #25, #28, #87, #94, #111) via supernatant culture and purified using Protein G affinity chromatography. Antigen-binding activity was assessed by indirect ELISA, in which plates were coated with recombinant A29L antigen at a concentration of 2 μg/mL.

**TABLE S2**. **Efficiency of mAbs biotin conjugation**.

| **Dilution** | **OD_450-620nm_ Value** | | | | | |
| --- | --- | --- | --- | --- | --- | --- |
|  | **Bio-5** | **Bio-25** | **Bio-28** | **Bio-87** | **Bio-94** | **Bio-111** |
| 1:2000 | 4.30 | 4.35 | 4.38 | 4.23 | 4.51 | 4.55 |
| 1:4000 | 4.60 | 4.34 | 4.49 | 4.09 | 4.56 | 4.18 |
| 1:8000 | 4.22 | 4.26 | 4.23 | 4.11 | 5.08 | 4.54 |
| 1:16000 | 4.45 | 4.07 | 4.13 | 3.84 | 4.27 | 3.97 |
| 1:32000 | 4.04 | 3.61 | 4.07 | 3.62 | 4.82 | 4.45 |
| 1:64000 | 3.81 | 2.82 | 3.13 | 2.81 | 4.09 | 4.02 |
| 1:128000 | 3.32 | 2.01 | 2.49 | 1.98 | 3.76 | 4.18 |
| Blank control | 0.02 | 0.03 | 0.02 | 0.02 | 0.02 | 0.02 |

Note: The biotin-conjugated derivatives of monoclonal antibodies MPXV-mAb#5, #25, #28, #94, and #111 are designated as Bio-5, Bio-25, Bio-28, Bio-94, and Bio-111, respectively. The antigen-binding activity of each conjugate was assessed by indirect ELISA using plates coated with recombinant A29L antigen at 2.0 μg/mL.

**TABLE S3**. **Results of monoclonal antibody pairwise screening**.

| **HRP-antibody** | **P/N values corresponding to antibodies in each coating** | | | | | |
| --- | --- | --- | --- | --- | --- | --- |
|  | **mAb #5** | **mAb #25** | **mAb #28** | **mAb #87** | **mAb #94** | **mAb #111** |
| Bio-5 | 3.50 | 5.48 | **28.6** | 6.45 | **25.4** | 6.19 |
| Bio-25 | 3.69 | 4.95 | 34.8 | 5.07 | 15.8 | 3.75 |
| Bio-28 | 26.5 | 20.0 | 24.5 | 18.2 | 11.9 | 7.62 |
| Bio-87 | 5.66 | 5.54 | 23.7 | 5.25 | 4.24 | 2.93 |
| Bio-94 | 23.6 | 17.4 | **25.8** | 15.0 | 12.5 | 7.06 |
| Bio-111 | 14.6 | 9.21 | 4.20 | 11.4 | 5.37 | 3.28 |

Note: Values that are underlined and bolded indicate the optimal pairing of coating antibody and enzyme-labeled antibody. Wells containing antigen diluent alone served as the negative control. The P/N value, defined as the ratio of the OD value of the test well to that of the negative control well, was used to determine positivity: samples with P/N ≥ 2.1 were considered positive, while those with P/N < 2.1 were considered negative.

**TABLE S4**. **Optimization of working concentrations for coating and HRP-conjugated antibodies.**

| Concentration of the coating antibodies  (mAb #94) | P/N values corresponding to paired mAbs | | | |
| --- | --- | --- | --- | --- |
|  | 1.00 μg/ml  (Bio-5) | 0.50 μg/ml  (Bio-5) | 0.20 μg/ml  (Bio-5) | 0.10 μg/ml  (Bio-5) |
| 2.00 μg/ml | ≥4.0/1.35 | ≥4.0/0.82 | ≥4.0/0.33 | **2.59/0.09** |
| 1.00 μg/ml | ≥4.0/0.50 | ≥4.0/0.22 | 2.35/0.14 | 1.04/0.06 |
| 0.50 μg/ml | 1.50/0.24 | 0.67/0.15 | 0.45/0.07 | 0.13/0.06 |
| 0.25 μg/ml  Note: The optimized working concentrations were 2.0 μg/mL for the coating antibody and 0.2 μg/mL for the HRP-conjugated antibody. | 0.71/0.13 | 0.34/0.08 | 0.14/0.04 | 0.08/0.03 |

**Table S5**. **Clinical evaluation of the commercially available ELISA kits using patient samples**.

| **Sample** | **OD_450-620nm_ Value** | | |
| --- | --- | --- | --- |
|  | **Plasma** | **Pharyngeal swab** | **Acne liquid** |
| Sample#1  Sample#2  Sample#3  Sample#4  Sample#5  Sample#6  Sample#7  Sample#8  Sample#9  Sample#10  Positive control  Negative control | 0.05  0.02  0.01  0.06  0.01  **0.13**  0.06  0.07  0.03  0.01  1.73  0.05 | 0.04  0.03  0.06  0.04  0.02  0.02  0.01  0.02  0.03  0.04  1.94  0.04 | 0.01  0.03  0.02  0.04  0.02  0.06  0.01  0.01  0.05  0.05  1.49  0.04 |

Note: Samples were considered positive when indicated by underlined and bolded values. Recombinant A29L antigen was used as the positive control. Negative controls consisted of samples from healthy donors for plasma and pharyngeal swabs, and PBS for vesicular fluid. A P/N value ≥ 2.1 was defined as the threshold for positivity.

**Table S6**. **The qPCR Ct values for all antigen-negative and positive samples from LFIA**

| **Samples** | **qPCR** **Positive\****Negative** | **qPCR Ct Value** | **LFIA Positive\Negative** |
| --- | --- | --- | --- |
| Patient 1 | Negative | - | Negative |
| Patient 2 | Positive | 38.8 | Negative |
| Patient 3 | Negative | - | Negative |
| Patient 4 | Positive | 25.7 | Positive |
| Patient 5 | Positive | 36.7 | Negative |
| Patient 6 | Positive | 23.2 | Positive |
| Patient 7 | Positive | 17.3 | Positive |
| Patient 8 | Positive | 18.7 | Positive |
| Patient 9 | Positive | 34.2 | Negative |
| Patient 10 | Negative | - | Negative |
| Patient 11 | Positive | 24.7 | Positive |
| Patient 12 | Positive | 22.6 | Positive |
| Patient 13 | Negative | - | Negative |
| Patient 14 | Positive | 38.8 | Negative |
| Patient 15 | Positive | 25.3 | Positive |
| Patient 16 | Positive | 23.2 | Positive |
| Patient 17 | Positive | 19.6 | Positive |
| Patient 18 | Positive | 35.5 | Negative |
| Patient 19 | Positive | 36.5 | Negative |
| Patient 20 | Negative | - | Negative |
| Patient 21 | Positive | 27.8 | Positive |
| Patient 22 | Positive | 29.6 | Positive |

Note: qPCR was performed using the commercially available "Monkeypox Virus Nucleic Acid Detection Kit (Fluorescent PCR Method)" from DAAN GENE (Guangzhou, China; Cat# 20243401527). The procedures and interpretation followed the manufacturer's instructions strictly.
