# Supplementary material for: Development and preliminary clinical validation of A29L mAb-based ELISA and lateral flow immunoassays for monkeypox virus detection
Source: Microbiol Spectr. 2026 Jun 15;14(7):e00167-26. doi: 10.1128/spectrum.00167-26 (PMC13340043; doi:10.1128/spectrum.00167-26)
Supplement: Reviewer comments [file reviewer-comments.pdf]

AMERICAN SOCIETY FOR MICROBIOLOGY | Microbiology Spectrum

Development and Preliminary Clinical Validation of High-Performance A29L mAb-Based Assays for Monkeypox Virus Detection

Jieguang Liu, Xujuan Hu, Qiong Chen, Xianglian Xiong, Yingjie Zhang, Leyiyi Liu, Si Du, Yuanjun Wu, Dan Fan, Jia Lu, Lianguo Ruan, and Fuli Ren

Corresponding Author(s): Fuli Ren, Chinese Academy of Sciences Wuhan Institute of Virology

Review Timeline:

Submission Date:	January 14, 2026
Editorial Decision:	February 8, 2026
Revision Received:	April 6, 2026
Accepted:	April 13, 2026

Editor: Ping Ren

Reviewer(s): Disclosure of reviewer identity is with reference to reviewer comments included in decision letter(s). The following individuals involved in review of your submission have agreed to reveal their identity: Fang Huang (Reviewer #1); Bahram Alamdary Badlou (Reviewer #2)

Transaction Report:

(Note: With the exception of the correction of typographical or spelling errors that could be a source of ambiguity, letters and reports are not edited. The original formatting of letters and referee reports may not be reflected in this compilation.)

DOI: https://doi.org/10.1128/spectrum.00167-26

Re: Spectrum00167-26 (Development and Clinical Validation of High-Performance A29L mAb-Based Assays for Monkeypox Virus Detection)

Dear Dr. Fuli Ren:

Thank you for the privilege of reviewing your work. Below you will find my comments, instructions from the Spectrum editorial office, and the reviewer comments.

Please return the manuscript within 60 days; if you cannot complete the modification within this time period, please contact me. If you do not wish to modify the manuscript and prefer to submit it to another journal, notify me immediately so that the manuscript may be formally withdrawn from consideration by Spectrum.

Revision Guidelines
To submit your modified manuscript, log into the submission site at https://spectrum.msubmit.net/cgi-bin/main.plex. Go to Author Tasks and click the appropriate manuscript title to begin. The information you entered when you first submitted the paper will be displayed; update this as necessary. Note the following requirements:

• Upload point-by-point responses to the issues raised by the reviewers in a file named "Response to Reviewers," NOT in your cover letter.
• Upload a compare copy of the manuscript (without figures) as a "Marked-Up Manuscript" file.
• Upload a clean .DOC/.DOCX version of the revised manuscript and remove the previous version.
• Each figure must be uploaded as a separate, editable, high-resolution file (TIFF or EPS preferred), and any multipanel figures must be assembled into one file.
• Any supplemental material intended for posting by ASM should be uploaded with their legends separate from the main manuscript. You can combine all supplemental material into one file (preferred) or split it into a maximum of 10 files with all associated legends included.

For complete guidelines on revision requirements, see our Submission and Review Process webpage. Submission of a paper that does not conform to guidelines may delay acceptance of your manuscript.

Data availability: ASM policy requires that data be available to the public upon online posting of the article, so please verify all links to sequence records, if present, and make sure that each number retrieves the full record of the data. If a new accession number is not linked or a link is broken, provide Spectrum production staff with the correct URL for the record. If the accession numbers for new data are not publicly accessible before the expected online posting of the article, publication may be delayed; please contact production staff (Spectrum@asmusa.org) immediately with the expected release date.

Publication Fees: For information on publication fees and which article types are subject to charges, visit our website. If your manuscript is accepted for publication and any fees apply, you will be contacted separately about payment during the production process; please follow the instructions in that e-mail. Arrangements for payment must be made before your article is published.

ASM Membership: Corresponding authors may join or renew ASM membership to obtain discounts on publication fees. Need to upgrade your membership level? Please contact Customer Service at Service@asmusa.org.

The ASM Journals program strives for constant improvement in our submission and publication process. Please tell us how we can improve your experience by taking this quick Author Survey.

Thank you for submitting your paper to Spectrum.

Sincerely,
Ping Ren
Editor
Microbiology Spectrum

Reviewer #1 (Comments for the Author):

Liu et al. generated a panel of monoclonal antibodies targeting A29L of monkeypox virus, and developed a highly sensitive enzyme-linked immunosorbent assay (ELISA) kit and a rapid lateral flow immunoassay (LFIA) for the detection of monkeypox virus antigen. First, based on the A29L protein of monkeypox virus, the researchers expressed A29L in vitro, immunized mice, screened antibodies by hybridoma technology, and verified the neutralizing activity of the antibodies. A total of 6 strains of

antibodies were screened out, among which 1 strain was a neutralizing antibody and the other 5 strains were binding antibodies. Finally, through various antibody pair combinations, they identified the most suitable antibody pairs for ELISA and colloidal gold detection, and tested their sensitivity and accuracy in clinical samples.

The study design is scientific and reasonable, and the experimental logic is rigorous. However, there are some minor issues that need to be supplemented and improved, as follows:

1.Monkeypox virus has subtypes such as West African clade and Congo Basin clade, and there is a possibility of genetic variation. The A29L protein sequence used in this study is derived from Clade II; it is necessary to supplement the sequence conservation of this A29L protein among different monkeypox virus strains/subtypes. In addition, it should be clarified whether the used A29L protein is the full-length protein or a specific fragment.

2.For the methodological part of the ELISA assay, it is recommended to report the LOD, linear detection range, and to add the evaluation results of interference experiments with different detection matrices (such as serum) to further verify the applicability of the method.

3.In the specificity verification of the two detection methods (ELISA and LFIA), it is not clearly stated whether the cross-reactivity of the methods with other related viruses in the Poxviridae family and other viruses (such as VZV, HSV, etc.) was detected. It is recommended to supplement the relevant verification experiments or give a detailed explanation.

4.Regarding the early diagnostic efficacy for patients in the acute phase of monkeypox, this study does not clearly illustrate the detection effect of the two methods in the early stage of the acute phase. In addition, for the samples that were not detected as positive (line 345), it is necessary to supplement their corresponding real-time fluorescent quantitative PCR (qPCR) Ct values to further analyze the sensitivity limitations of the detection methods.

5.In the text description of the Result section (line 346), the corresponding figure should be Figure 5. Please check and correct the figure number labeling error.

Reviewer #2 (Comments for the Author):

Although the authors presented very important scientific issue but their main text contains too many vague words and phrases, resulting in a manuscript that is not comprehensive and clear for readers. i.e. in the whole main text abusing words like.... this 33x, that... 17x and those... 10x times. Moreover, according to title ...this a validation study but ...Pape 14 lines 410-212 mentioned The present study is not without its limitations. Firstly, clinical validation was "conducted on a limited", single-center cohort from Hubei Province, China (n=22 ????. ...page14 lines 417-418 It is recommended that future multicenter studies be conducted, employing larger
sample sizes to further validate?????????

Point-by-Point Responses to Reviewers

Response to Reviewer #1:

We sincerely thank the reviewer for the positive evaluation of our study's design and for providing these expert and constructive suggestions, which have greatly helped us improve the manuscript.

Comment 1:
Monkeypox virus has subtypes such as West African clade and Congo Basin clade, and there is a possibility of genetic variation. The A29L protein sequence used in this study is derived from Clade II; it is necessary to supplement the sequence conservation of this A29L protein among different monkeypox virus strains/subtypes. In addition, it should be clarified whether the used A29L protein is the full-length protein or a specific fragment.
Response 1:
We thank the reviewer for raising this important point. To address it, we have supplemented a sequence conservation analysis in the Discussion section, confirming a high degree of amino acid identity (>99%) for A29L among the major MPXV clades (I (Congo Basin), IIa, IIb (West African)), which supports the potential broad applicability of our antibodies. Furthermore, we have explicitly clarified in both the Materials and Methods ("Recombinant Protein Production and Animal Immunization") and the Results ("Recombinant Antigen Production, Immunization, and Hybridoma Generation) sections that the immunogen and assay standard used throughout this study was the full-length A29L gene expressed as a GST-fusion protein (A29L-GST).

Comment 2:
For the methodological part of the ELISA assay, it is recommended to report the LOD, linear detection range, and to add the evaluation results of interference experiments with different detection matrices (such as serum) to further verify the applicability of the method.
Response 2:
We sincerely thank the reviewer for this critical and constructive suggestion, which underscores the importance of rigorous methodological reporting and validation for real-world applicability. We have comprehensively addressed this point in the revised manuscript.
Regarding the systematic reporting of the LOD and linear range: We fully agree that these are fundamental parameters. In the revised MATERIALS AND METHODS section (subsection "Development of the Sandwich ELISA"), we have now added a dedicated statement that explicitly lists the assay's limit of detection (LOD: 31.25 pg/mL), limit of quantification (LOQ: 62.50 pg/mL), and linear quantitative range (31.25–2000 pg/mL). These values are also clearly reiterated in the Results section ("Analytical Performance of the Optimized Sandwich ELISA") and in Table 2, ensuring consistency and prominence.

Regarding the evaluation of matrix interference: We appreciate the reviewer's emphasis on verifying assay robustness in complex matrices. Our ELISA was optimized and calibrated using recombinant A29L antigen spiked in the assay diluent (1% BSA/PBS), which provides the baseline analytical performance in a controlled matrix. Guided by the clinical context of our study, we focused on the most relevant sample types for MPXV diagnosis. It is important to note that, consistent with the pathobiology of MPXV, systemic viremia is typically low-level or transient, whereas peak viral loads are found in lesion-derived samples and anal swabs. Therefore, while serum is a common diagnostic matrix, its utility for MPXV antigen detection is limited. Consequently, we prioritized the direct clinical evaluation of our assay in the most relevant and challenging patient matrices: plasma, vesicular fluid, and swabs. The excellent sensitivity (100%) and specificity achieved in patient plasma samples (Table 3) serve as a direct and robust demonstration of the assay's functionality in this complex biological matrix from infected individuals. This clinical validation in real-world samples provides strong evidence of applicability that complements.

In summary, we have strengthened the manuscript by fully reporting the key analytical parameters. Regarding matrix effects, we have clarified our validation strategy, which is grounded in the specific pathobiology of MPXV and culminates in direct clinical testing, thereby providing a compelling verification of the assay's practical utility.

Comment 3:

In the specificity verification of the two detection methods (ELISA and LFIA), it is not clearly stated whether the cross-reactivity of the methods with other related viruses in the Poxviridae family and other viruses (such as VZV, HSV, etc.) was detected. It is recommended to supplement the relevant verification experiments or give a detailed explanation.

Response 3:

We thank the reviewer for raising this critical point regarding assay specificity, which has allowed us to clarify and highlight the comprehensive validation already conducted.

In response, we wish to clarify that the requested specificity evaluations were in fact performed and are presented in the manuscript. First, as now explicitly stated in the Materials and Methods section ("Virus, Antigens and Clinical Specimens"), we included and tested the A27L antigen of vaccinia virus (VACV), a closely related orthopoxvirus and a common source of cross-reactivity, in our specificity panel. Second, our experimental validation extended beyond poxviruses to also include varicella-zoster virus (VZV) and herpes simplex virus (HSV) antigens.

The results of all these specificity tests are clearly reported in the Results section ("Analytical Performance of the Optimized Sandwich ELISA"), demonstrating no appreciable cross-reactivity with VACV, VZV, or HSV (as part of the data summarized in Figure 3).

Comment 4:

Regarding the early diagnostic efficacy for patients in the acute phase of monkeypox, this study does not clearly illustrate the detection effect of the two methods in the early stage of the acute phase. In addition, for the samples that were not detected as positive (line 345), it is necessary to supplement their corresponding real-time fluorescent quantitative PCR (qPCR) Ct values to further analyze the sensitivity limitations of the detection methods.

Response 4:

We are grateful for this suggestion, which allowed for a more insightful clinical performance analysis.

In response, we performed a re-analysis correlating our antigen detection results with qPCR Ct values. This analysis reveals a key finding: our lateral flow immunoassay (LFIA) achieved 100% sensitivity in samples with high viral loads (qPCR Ct < 30), which are characteristic of the acute and most contagious phase of infection. This clearly defines the LFIA's primary role for rapid frontline screening. The qPCR Ct values for all antigen-negative and positive samples from LFIA are now provided in the relevant part of the Results ("Clinical Evaluation of the ELISA and LFIA") and in Supplementary Table S6, objectively illustrating the sensitivity boundaries of our tests.

Comment 5:

In the text description of the Result section (line 346), the corresponding figure should be Figure 5. Please check and correct the figure number labeling error.

Response 5:

We thank the reviewer for the careful reading.

We have thoroughly checked and corrected the figure citation errors (specifically, the confusion between Figure 4 and Figure 5) throughout the manuscript. All in-text citations now correctly match the corresponding figure numbers.

Response to Reviewer #2:

We thank the reviewer for the critical feedback regarding language clarity and the scope of validation. These comments have been invaluable in enhancing the precision and readability of our manuscript.

Comment:

Although the authors presented very important scientific issue but their main text contains too many vague words and phrases, resulting in a manuscript that is not comprehensive and clear for readers. i.e. in the whole main text abusing words like.... this 33x, that... 17x and those... 10x times. Moreover, according to title ...this a validation study but ...Pape 14 lines 410-212 mentioned The present study is not without its limitations. Firstly, clinical validation was "conducted on a limited", single-center cohort from Hubei Province, China (n=22 ????. ...page14 lines 417-418 It is recommended that future multicenter studies be conducted, employing larger sample sizes to further validate?????????

Response:

We sincerely thank you for the meticulous review and the highly valuable critique. The issues raised regarding linguistic clarity and logical consistency are crucial for improving the quality of our manuscript. We fully acknowledge these points and have undertaken comprehensive revisions in response.

1) Regarding the overuse of vague referents (this, that, those): We have systematically revised the entire manuscript to address this concern. Each instance of ambiguous reference has been examined. Where possible, vague pronouns have been replaced with specific nouns or descriptive phrases, and sentence structures have been rephrased for clarity. This extensive edit enhances the precision and readability of the Abstract, Introduction, Materials and Method, Results, and Discussion sections, ensuring all descriptions and conclusions are unequivocally clear to the reader.
2) Regarding the study's scope and the conclusion's logic: We appreciate the reviewer's point on logical rigor. We have also revised the article title to "Development and Preliminary Clinical Validation of High-Performance A29L mAb-Based Assays for Monkeypox Virus Detection" to accurately reflect the preliminary nature of the clinical evaluation. This change aligns the title with the refined description of the study's scope and conclusions within the manuscript. The core of this work is the development and preliminary clinical evaluation of a diagnostic toolkit (mAbs, ELISA, LFIA) for MPXV, not a definitive, large-scale multicenter clinical validation trial. To clarify this and align the narrative:
    a. In the concluding paragraph, we have reframed the discussion of limitations. Instead of a generic "limitations" section, we now state: "The impact of this study is constrained by the preliminary nature of the clinical evaluation, which was conducted on a limited, single-center cohort…" This more accurately reflects the study phase.

b. We have revised the final recommendation. It now reads as a forward-looking statement consistent with a development study: “To ultimately translate these assays into widespread clinical use, future multicenter studies with larger sample sizes are required to validate their utility across diverse populations and MPXV clades.” This positions future work as the necessary next step for translation, not as an admission that the current study’s own aims were unfulfilled.

We believe these revisions have substantially improved the manuscript's clarity, logic, and academic rigor. We are grateful for the reviewer's insightful comments.

Re: Spectrum00167-26R1 (**Development and Preliminary Clinical Validation of High-Performance A29L mAb-Based Assays for Monkeypox Virus Detection**)

Dear Dr. Fuli Ren:

Your manuscript has been accepted, and I am forwarding it to the ASM production staff for publication. Your paper will first be checked to make sure all elements meet the technical requirements. ASM staff will contact you if anything needs to be revised before copyediting and production can begin. Otherwise, you will be notified when your proofs are ready to be viewed.

Data Availability: ASM policy requires that data be available to the public upon online posting of the article, so please verify all links to sequence records, if present, and make sure that each number retrieves the full record of the data. If a new accession number is not linked or a link is broken, provide production staff with the correct URL for the record. If the accession numbers for new data are not publicly accessible before the expected online posting of the article, publication may be delayed; please contact ASM production staff immediately with the expected release date.

Publication Fees: For information on publication fees and which article types have charges, please visit our website. We have partnered with Copyright Clearance Center (CCC) to collect author charges. If fees apply to your paper, you will receive a message from no-reply@copyright.com with further instructions. For questions related to paying charges through RightsLink, please contact CCC at ASM_Support@copyright.com or toll free at +1-877-622-5543. CCC makes every attempt to respond to all emails within 24 hours.

ASM Membership: Corresponding authors may join or renew ASM membership to obtain discounts on publication fees. Need to upgrade your membership level? Please contact Customer Service at Service@asmusa.org.

Embargo Policy: A press release may be issued as soon as the manuscript is posted on the Spectrum Latest Articles webpage. The corresponding author will receive an email with the subject line "ASM Journals Author Services Notification" when the article is available online.

The ASM Journals program strives for constant improvement in our submission and publication process. Please tell us how we can improve your experience by taking this quick Author Survey.

Thank you for submitting your paper to Spectrum.

Sincerely,
Ping Ren
Editor
Microbiology Spectrum
